# Impact of the National Health Insurance Coverage Policy on the Utilisation and Accessibility of Innovative Anti-cancer Medicines in China: An Interrupted Time-Series Study

**DOI:** 10.3389/fpubh.2021.714127

**Published:** 2021-08-06

**Authors:** Wenqing Fang, Xinglu Xu, Yulei Zhu, Huizhen Dai, Linlin Shang, Xin Li

**Affiliations:** ^1^Department of Public Health, School of Health Policy and Management, Nanjing Medical University, Nanjing, China; ^2^Department of Clinical Pharmacy, School of Pharmacy, Nanjing Medical University, Nanjing, China; ^3^Jiangsu Medicine Information Institute, Nanjing, China; ^4^Center for Global Health, Nanjing Medical University, Nanjing, China

**Keywords:** innovative anti-cancer medicines, national health insurance coverage, utilisation, affordability, interrupted time series

## Abstract

**Objective:** The study aimed to evaluate the impact of the National Health Insurance Coverage (NHIC) policy on the utilisation and accessibility of innovative anti-cancer medicines in Nanjing, China.

**Methods:** We used the adjusted World Health Organisation and Health Action International methodology to calculate the price and availability of 15 innovative anti-cancer medicines included in the National Health Insurance drug list in 20 tertiary hospitals and six secondary hospitals in Nanjing before and after NHIC policy implementation. Interrupted time-series regression was used to analyse the changes in the utilisation of the study medicines.

**Results:** The price reduction rates of innovative anti-cancer medicines ranged between 34 and 65%. The mean availability rate was 27.44% before policy implementation and increased to 47.33% after policy implementation. The utilisation of anti-cancer medicines suddenly increased with a slope of 33.19–2,628.39 when the policy was implemented. Moreover, the usage rate of bevacizumab, bortezomib, and apatinib significantly increased (*p* < 0.001, *p* = 0.009, and *p* < 0.001, respectively) after policy implementation. With regard to price reduction and medical insurance reimbursement, the medicines became more affordable after policy implementation (0.06–1.90 times the per capita annual disposable income for urban patients and 0.13–4.46 times the per capita annual disposable income for rural patients).

**Conclusion:** The NHIC policy, which was released by the central government, effectively improved the utilisation and affordability of innovative anti-cancer medicines. However, the availability of innovative anti-cancer medicines in hospitals remained low and the utilisation of innovative anti-cancer medicines was affected by some factors, including the incidence of cancer, limitation of indications within the insurance program, and the rational use of innovative anti-cancer medicines. It is necessary to improve relevant supporting policies to promote the affordability of patients. The government should speed up the process of price negotiation to include more innovative anti-cancer medicines in the medical insurance coverage, consider including both medical examinations and adjuvant chemotherapy in the medical insurance, and increase investment in health care.

## Introduction

Cancer has been a major health issue worldwide and is one of the most serious health problems in China. Based on the data from the National Central Cancer Registry in 2019, 2.929 million patients were newly diagnosed with cancer and 2.338 million deaths were reported (1). Cancer accounts for 25% of all cases and deaths worldwide (2). Population growth and increased life expectancy are among the important reasons for the high morbidity of cancer (2).

To address the increase in the risk of cancer, the pharmaceutical industry has focused on the research and development of novel anti-cancer medicines worldwide. In recent years, a number of innovative anti-cancer medicines were approved for marketing every year (3), such as molecular targeted therapy drugs (osimertinib), immunotherapy drugs, programmed cell death protein (PD)-1 inhibitors, and PD ligand 1 inhibitors. Innovative anti-cancer medicines play an important role in the treatment of malignant tumours, bringing hope to patients with cancer who have a higher mortality risk. Moreover, innovative anti-cancer medicines can improve the quality of life; some patients may even achieve normal lives (4). Opinions of the State Council on the Implementing the Healthy China Action clearly stated that by 2022 and 2030, the overall 5-year survival rates of cancer should be >43.3% and >46.6%, respectively (5). Actively advocating for cancer prevention and promoting early diagnosis and treatment, supplemented by the early use of suitable innovative anticancer medicines, can help patients receive better treatment, thereby improving the overall five-year survival rate (6). However, due to patent protection and technology monopoly of innovative drugs, the cost of innovative anti-cancer medicines is usually higher than the catastrophic health expenditure, which imposes a financial burden on patients and hinders access to these medicines (7, 8).

In some developing countries, the financing healthcare systems rely mainly on out-of-pocket payment (OOP), which increases the risk of financial catastrophe in many patients with cancer and their families (9). In China, the high cost of cancer treatment has become a major issue in the area of social security. An empirical study conducted by the National Cancer Centre in 2016 showed that the treatment cost per patient with cancer in China was US$9,739, while the average household income in the same year was US$8,607 (10). Another study in inner Mongolia found that the annual direct medical cost for cancer reached CNY 86,100 after the basic medical insurance reimbursement, which exceeded the “catastrophic health expenditure” defined by the World Health Organisation (WHO) and imposed a heavy economic and mental burden on patients and their families (11). Furthermore, in China, a 1-month supply of Gleevec costs > CNY 23,000, which is not covered by health insurance in most cities in China. A patient with leukaemia named Yong Lu borrowed the idea from the Oscar-winning film *Dallas Buyers Club* and smuggled unapproved, India-made drugs for himself and for >1,000 other patients to obtain medicines at a relatively affordable price. However, any unapproved medications are illegal in China (12). Patients often give up treatment because of financial burden, putting their physical and mental health at risk (13).

In low- and middle-income countries (LMICs), poor availability and high expenditure are among the greatest obstacles to cancer treatment (14). Even in developed countries such as the United States, 29% of cancer survivors reported at least one cancer-related financial problem (15). Statistics have shown that during the median survival period, most cancer patients spend >US$150,000. In fact, only a few patients used innovative anti-cancer medicines. In 2017, 87% of these drugs were used in < 1,000 patients nationwide (16). It is important to guarantee the availability and affordability of anti-cancer medicines (17) to measure whether patients can receive medical treatment at an affordable price (18).

The 2011 Political Declaration of the High-Level Meeting of the United Nations General Assembly on the Prevention and Control of Non-Communicable Diseases declared that the global burden and threat of non-communicable diseases including cancer have caused a great social and economic burden. It highlighted the primary role of governments and the responsibilities they need to undertake (19). Thus, to improve the rational use of drugs and promote accessibility and affordability of innovative anti-cancer medicines, the Chinese government implemented a series of policies such as the National Health Insurance Coverage (NHIC) policy for innovative medicines.

For instance, Yang et al. calculated the financial burden of patients with human epidermal growth factor receptor 2-positive breast cancer who used trastuzumab after the implementation of special insurance drug coverage in Jiangsu province. The results showed that the total medical cost had dropped significantly compared to the price before policy implementation. Moreover, the compensation ratio of reimbursement increased from 23.44 to 72.81% after policy implementation (20). Tian et al. evaluated the affordability of three anti-cancer targeted drugs (gefitinib, trastuzumab, and sunitinib) used in urban and rural residents of Hubei province using the WHO and the Health Action International (HAI) standard survey method. The affordability of the three drugs increased from 64 to 74% after applying a 50% discount and the reimbursement (21). On the other hand, Diao et al. used an interrupted time-series (ITS) design to evaluate the changes in the utilisation of targeted anti-cancer medicines covered by the provincial government health insurance program from 2013 to 2016 in 69 hospitals in Hangzhou, Zhejiang province. The results showed that the inclusion of expensive targeted anti-cancer medicines in government health insurance coverage notably increased the utilisation of these medicines and improved the affordability of patients. However, the financial burden of patients remained high (22).

In April 2017, the Ministry of Human Resources and Social Security of the People's Republic of China initiated the centralised strategic price negotiation with pharmaceutical companies, which gave full play to the advantage of “group purchase” in terms of exchange quantity and price. After the price negotiation, the prices of innovative medicines were reduced and some new medicines were included in the National Insurance Medicine List. Moreover, the pharmaceutical enterprises obtained the market share under the premise of ensuring their business profits (23, 24). A total of 44 new medicines were included in the price negotiation, and a reduction in the price of 36 medicines was successfully negotiated. On average, the price of innovative medicines was reduced by 44%. Among the 36 new medicines, 15 innovative anti-cancer medicines were included in the “National Basic Medical Insurance, Work-Related Injury Insurance and Childbirth Insurance Medicine List (Category B)” (25), which was an important step toward facilitating access to expensive anti-cancer medicines (26).

The NHIC policy has been implemented in Nanjing, Jiangsu province since September 1, 2017. The 15 negotiated innovative anti-cancer medicines were listed in the “Provincial Medical Institution Drugs Centralised Procurement Online and Supervision Platform,” along with the medical insurance payment standards. With this, all public medical institutions in Nanjing could purchase the innovative anti-cancer medicines on the centralised platform. Furthermore, to reduce the financial burden of patients with cancer, the local health insurance policy reimbursed 70–90% of the cost of the 15 innovative anti-cancer medicines in Nanjing.

However, there was little evidence for government about the changes in the availability, utilisation, and affordability of these 15 anti-cancer medicines after policy implementation. Thus, in this study, we tried to explore whether the NHIC policy had a positive impact on the price, availability, utilisation, and affordability of anti-cancer medicines in Nanjing City.

Using the WHO/HAI methodology, the study aimed to analyse the impact of the NHIC policy on the availability and affordability of negotiated innovative anti-cancer medicines in 20 tertiary hospitals and six secondary hospitals in Nanjing City, the capital city of Jiangsu province. Furthermore, an ITS analysis was used to analyse the changes in the utilisation of the study medicines.

## Materials and Methods

### Study Design

The study was performed in Nanjing City from January 2016 to December 2018. Nanjing is the capital of Jiangsu province. It has 11 municipal districts and a population of >6.8 million permanent residents. The gross domestic product was CNY 1.17 trillion in 2017 (27). The NHIC policy was implemented on September 1, 2017, in Nanjing, where cancer is ranked as the second leading cause of death (28).

### Sample and Data Source

We analysed the monthly sales data of the surveyed anti-cancer medicines from January 2016 to December 2018, which were obtained from the Jiangsu Medicine Information Institute. As primary hospitals are not qualified to prescribe most anti-cancer medicines, they were not included among the study hospitals. Moreover, tertiary and secondary hospitals that did not provide oncotherapy services were also excluded due to the lack of tumour therapy services. Hence, 20 tertiary hospitals and six secondary hospitals, including an oncology department, were included in this study. A total of 15 innovative anti-cancer medicines were evaluated in this survey. The essential features of the 15 drugs are presented in Table 1.

**Table 1:** Baseline information of 15 innovative anti-cancer medicines.

**No**.	**Name**	**Dosage form**	**Strength**	**Indications**	**Pharmaceutical company**
1	Rituximab	Injection	100 mg/10 ml	Lymphoma	Roche
2	Trastuzumab	Injection	440 mg/20 ml	Breast cancer and gastric cancer	Roche
3	Bevacizumab	Injection	100 mg/4 ml	Colorectal cancer and lung cancer	Roche
4	Sorafenib	Tablet	200 mg	Kidney cancer, hepatocellular carcinoma, and thyroid cancer	Bayer
5	Bortezomib	Injection	13.5 mg	Mantle cell lymphoma	Hansoh, Johnson & Johnson
6	Erlotinib	Tablet	150 mg	Lung cancer	Roche
7	Nimotuzumab	Injection	50 mg/10 ml	Nasopharynx cancer	Biotech Pharma
8	Apatinib	Tablet	250, 425 mg	Gastric adenocarcinoma	Jiangsu Hengrui Pharmaceuticals
9	Chidamide	Tablet	5 mg	Peripheral T-cell lymphoma	Chipscreen Biosciences
10	Fulvestrant	Injection	250 mg/5 ml	Breast cancer	AstraZeneca
11	Everolimus	Tablet	5 mg	Kidney cancer	Novartis
12	Lenalidomide	Capsule	10, 25 mg	Multiple myeloma	Celgene
13	Lapatinib	Tablet	250 mg	Breast cancer	GlaxoSmithKline
14	Abiraterone	Tablet	250 mg	Prostate cancer	Johnson & Johnson
15	Recombinant human endostatin	Injection	15 mg/2.4 ×10^5^U/3 ml	Lung cancer	Simcere

### Date Analysis

#### Price

Price was expressed as the median defined daily dose cost (DDDc). A higher DDDc indicated that the medicine was more expensive. We calculated the DDDc by dividing the total amount of medicine into Defined Daily Doses (DDDs) before and after policy implementation.

#### Availability

Availability of innovative anti-cancer medicines was expressed as the proportion of all survey hospitals that had the study medicines within the time period. Furthermore, we compared the availability of these medicines between tertiary and secondary hospitals before and after policy implementation. The following criteria were used to describe the level of availability (18, 29, 30):

- Absent: 0%, the medicine was not available in any surveyed hospital;
- Very low: <30%, the medicine was available hardly in surveyed hospitals;
- Low: 30–50%, the medicine was available in few surveyed hospitals;
- Fairly high: 50–80%, the medicine was available in many surveyed hospitals;
- High: >80%, the medicine had a good availability and was available in most surveyed hospitals.

#### Utilisation

Utilisation was expressed as monthly DDDs; the greater the DDDs, the greater frequency of using the medicine. The monthly DDDs were calculated by dividing the monthly sales data in volume into the DDD. As there was no standard DDD for anti-cancer medicines, we accessed the medicine DDD information based on the daily dose and its main indication from the authoritative medicine specification database. ITS regression analysis was used to analyse the changes in the utilisation of anti-cancer medicines within 36 months. When it was difficult or impossible to find a control group, the ITS model considered a quasi-experimental design to analyse the longitudinal effects of the interventions. The ITS model could evaluate whether policy intervention had a transient or long-term impact (31). The NHIC policy was implemented on September 1, 2017. Hence, there were 20 months before intervention and 16 months after intervention. To perform independent tests, utilisation was divided into three parts—(i) the slope of the utilisation before policy implementation, (ii) the change in the level of policy intervention, and (iii) the slope after policy implementation. The following ITS model formula was used:

$$\begin{array}{l} Y_{t}=\beta_{0}+\beta_{1}T+\beta_{2}D+\beta_{3}P+\varepsilon \end{array}$$

**Figure 1:**
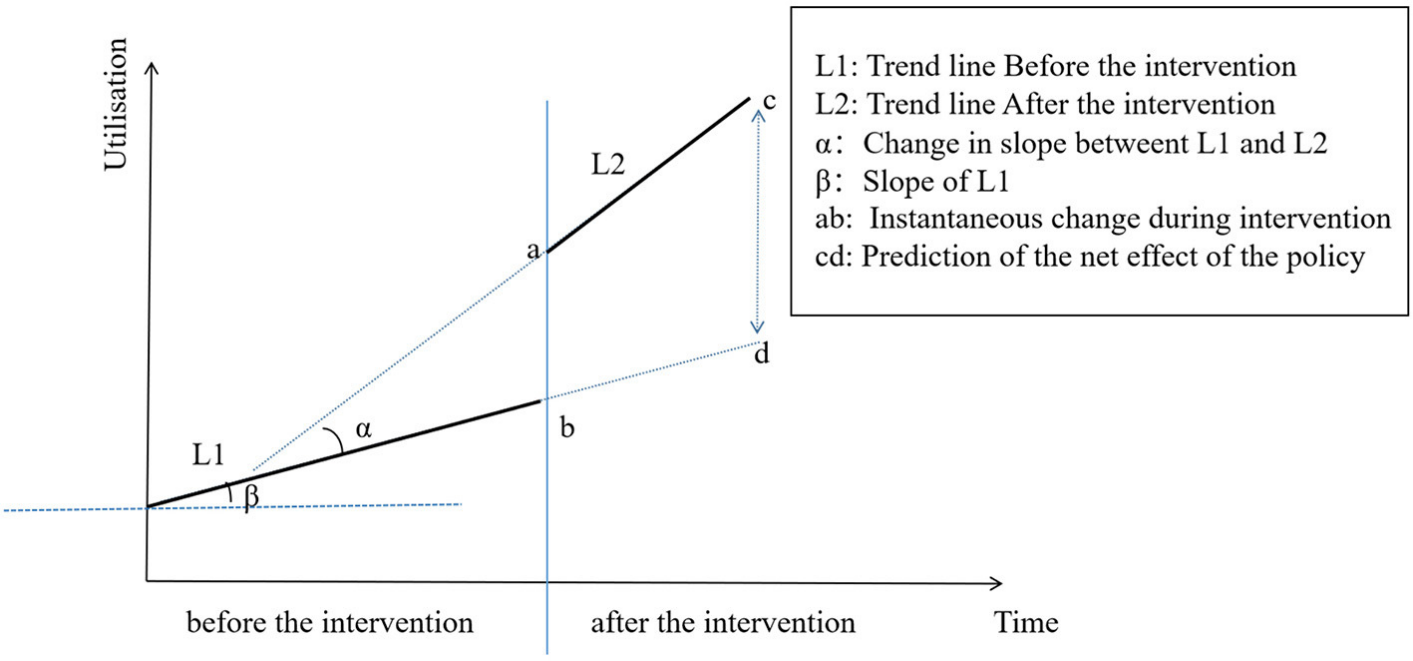
Graphic illustration of the interrupted time-series model and measurement of the policy effect based on trend lines for data points before and after policy implementation.

*Y*_t_ is the monthly utilisation measured at each *T* time point. *T* is the time since the study was initiated (*T* = 1, 2, 3 . 36). *D* is the dummy variable for the two time periods before and after policy implementation (*D* = 0 represents the time period before policy implementation and *D* = 1 represents the time period after policy implementation). *P* is the time point after policy intervention (*P* = 0 indicates before policy intervention and *P* = 1, 2, 3, 16 indicates after policy intervention). *β*_0_ is the intercept (which refers to the outcome when the time is 0 at the baseline level), *β*_1_ is the slope of the baseline, *β*_2_ is the level of changes in the intervention, *β*_3_ is the trend change of the outcome caused by the policy intervention, *β*_1_ + *β*_3_ is the slope after the intervention, and ε is the error term (32). The ITS model is presented in Figure 1.

The Durbin–Watson test was used to test the first-order autocorrelation of the data (33). It is extremely possible that the observations are independent. The feasible generalised least square method was used to modify the first-order autocorrelation errors if needed (34–36). All analyses were performed using STATA v.14 software (STATA Corporation, College Station, TX, USA), and a *p* = 0.05 was considered significant.

#### Affordability

Using the WHO/HAI affordability method, the affordability of patients was estimated using the minimum daily income of unskilled government workers by determining the daily income required to purchase the selected courses of treatment for a common acute or chronic condition. Generally, if the outcome was <1, the medicine was affordable for patients. If the outcome was >1, the patients could not afford the medicines. In consideration of the long-term treatment and heavy financial burden of anti-cancer medicines, a study in Hangzhou, Zhejiang province calculated the affordability as the number of average per capita disposable annual income needed to pay for the OOP expenditure of the study medicines based on the standard treatment guideline of a certain regimen used in patients within a particular duration (22). In this study, we assessed the duration of medicine treatment based on the median progression-free survival (mPFS). For trastuzumab, the 1-year mPFS was evaluate based on the treatment guideline (37–48).

OOP expenditure = the total cost of medicine × (1–proportion of reimbursement)

The proportion of reimbursement was based on each drug OOP ratio, defined by the Nanjing Medical Insurance Bureau.

Availability of patients = OOP expenditure of the medicine for mPFS treatment course/per capita annual disposable income

Data on the per capita annual disposable income were obtained from the 2018 and 2019 Nanjing Statistical Yearbook (27, 49).

Before policy implementation, some innovative anti-cancer medicines were included in the patient assistance program (PAP) of pharmaceutical companies. In China, PAP refers to the donation of drugs or funds by pharmaceutical companies to charitable organisations or other third-party, non-profit organisations and is initiated by these organisations to help patients with specific conditions. Patients with low income or a guaranteed minimum income as defined by the local government are included in the PAP. The eligible patients receive a defined free dose of innovative anti-cancer medicines after making an OOP of a defined dose. The six innovative anti-cancer medicines included in the PAP are shown in Table 2.

**Table 2:** Patient assistance program (PAP) of pharmaceutical companies to increase access to six innovative anti-cancer medicines.

**Name**	**Indication**	**PAP**
Trastuzumab	Breast cancer	8 vials paid by the PAP after 6 vials paid by patient
Bevacizumab	Colorectal cancer	Free after payment of 4 M
Sorafenib	Kidney cancer	Free after payment of 3 M
Bortezomib	Mantle cell lymphoma	Company donates 6 treatment courses after 3 treatment courses paid
Erlotinib	Non-small cell lung cancer	Free after payment of 4 M
Fulvestrant	Breast cancer	Company donates 3 M after 3 M paid

*PAP, patient assistance program*.

*M, Months*.

## Results

### Changes in the Price of Anti-cancer Medicines

Table 3 shows the changes in the DDDc of the study anti-cancer medicines. The rate of change shows the impact of the NHIC policy on the DDDc. As a result of the national price negotiation, the DDDc significantly decreased, varying from 34 to 65%. The price reduction rate was between 34.39 (recombinant human endostatin) and 65.45% (trastuzumab), and mean reduction rate was 50.1%.

**Table 3:** Prices of 15 innovative anti-cancer medicines before and after policy implementation (CNY).

**No**.	**Name**	**Before**	**After**	**Rate of change/%**
1	Rituximab	4,346.25	2,802.47	−35.52
2	Trastuzumab	999.97	345.45	−65.45
3	Bevacizumab	1,300.00	499.50	−61.58
4	Sorafenib	1,666.67	812.00	−51.28
5	Bortezomib	1,443.43	698.97	−51.58
6	Erlotinib	460.00	195.00	−57.61
7	Nimotuzumab	972.40	486.20	−50.00
8	Apatinib	731.34	462.40	−36.77
9	Chidamide	—	669.90	—
10	Fulvestrant	181.24	79.68	−56.04
11	Everolimus	495.00	296.00	−40.20
12	Lenalidomide	2,104.46	826.49	−60.73
13	Lapatinib	—	245.00	—
14	Abiraterone	—	579.68	—
15	Recombinant human endostatin	544.16	357.00	−34.39

“*—” means that this drug could not be available in this time period*.

### Changes in the Availability of Anti-cancer Medicines

As shown in Table 4, some anti-cancer medicines were unavailable before policy implementation. The mean availability of the studied anti-cancer medicines improved after policy implementation of medical insurance coverage (32.67 vs. 48.67% in tertiary hospitals; 10 vs. 25.56% in secondary hospitals). The availability of these medicines in tertiary hospitals was higher than that in secondary hospitals.

**Table 4:** Availability of 15 innovative anti-cancer medicines before and after policy implementation.

**No**.	**Name**	**Tertiary hospital/%**		**Secondary hospital/%**	
		**Before**	**After**	**Before**	**After**
1	Rituximab	85.00	85.00	50.00	33.33
2	Trastuzumab	70.00	50.00	33.33	50.00
3	Bevacizumab	45.00	85.00	33.33	83.33
4	Sorafenib	15.00	50.00	0.00	0.00
5	Bortezomib	40.00	45.00	0.00	33.33
6	Erlotinib	45.00	55.00	0.00	50.00
7	Nimotuzumab	30.00	40.00	0.00	0.00
8	Apatinib	40.00	55.00	0.00	33.33
9	Chidamide	0.00	20.00	0.00	0.00
10	Fulvestrant	10.00	60.00	0.00	16.67
11	Everolimus	20.00	25.00	0.00	0.00
12	Lenalidomide	10.00	35.00	0.00	0.00
13	Lapatinib	0.00	15.00	0.00	0.00
14	Abiraterone	0.00	35.00	0.00	33.33
15	Recombinant human endostatin	80.00	75.00	33.33	50.00

### Changes in the Level of Utilisation

Chidamide, fulvestrant, lapatinib, lenalidomide, lapatinib, and abiraterone were excluded as these medicines had no purchase records at most time points.

**Figure 2:**
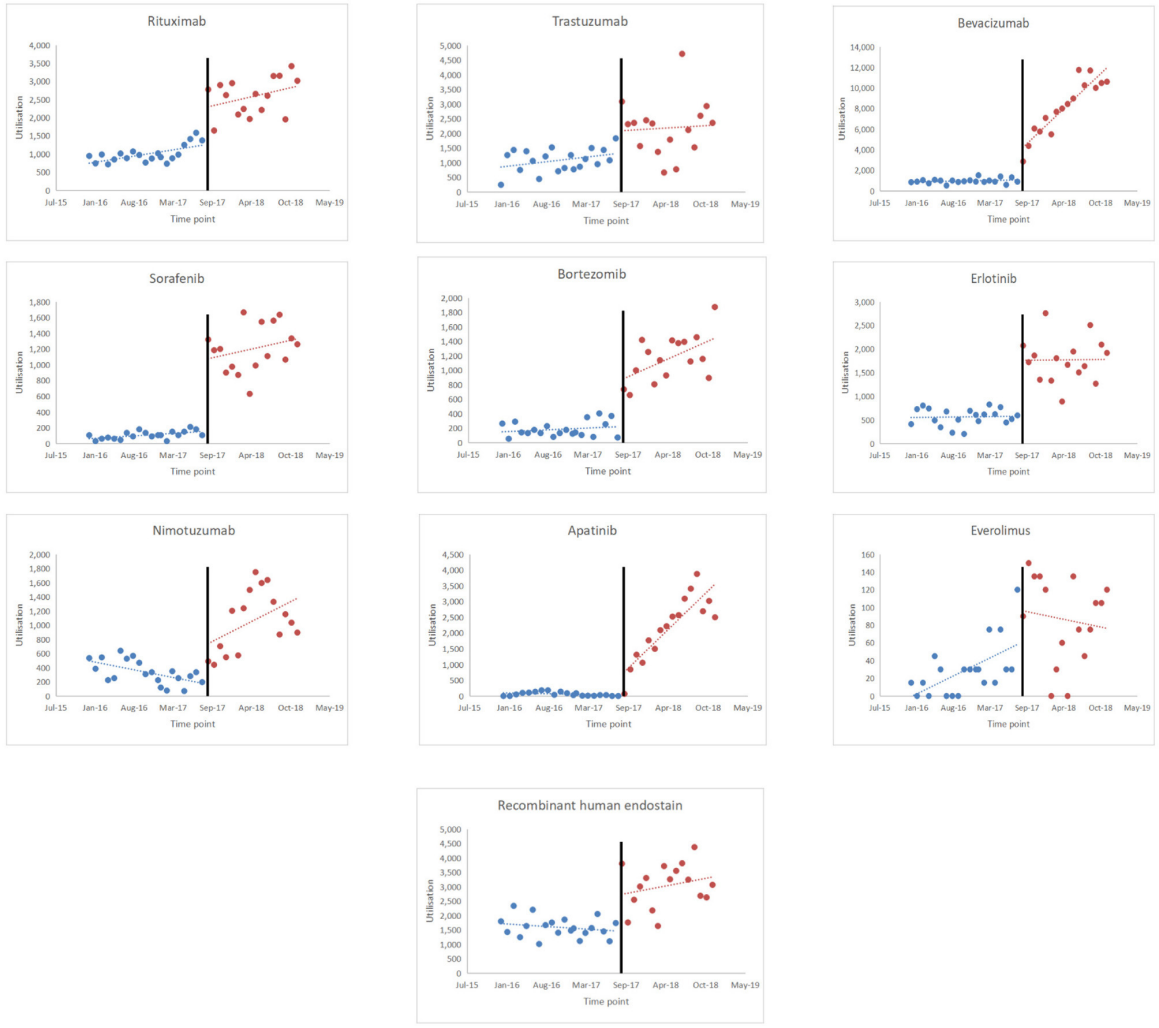
Results of the regression analysis of the utilisation of study drugs before and after policy implementation shown in a scatter plot.

Figure 2 shows the scatter plots of the observed utilisation of the study medicines. The implementation period of the policy intervention was considered September 1, 2017, and the time series was divided into two parts. Based on the results shown in Table 5, the utilisation of rituximab increased significantly before policy implementation (*p* < 0.001). When the NHIC policy was implemented, the utilisation rate suddenly increased [range: 33.19 (*p* = 0.247, everolimus) to 2,628.39 (*p* < 0.001, bevacizumab)]. The utilisation rate of bevacizumab, bortezomib, and apatinib significantly increased (*p* < 0.05), while that of trastuzumab, erlotinib, and everolimus decreased, with no significant difference (*p* = 0.817, *p* = 0.973, and *p* = 0.152, respectively). An upward trend was observed in the utilisation rate of nimotuzumab and recombinant human endostatin.

**Table 5:** Changes in the trend and level of utilisation estimated by ITS regression analysis.

**No**.	**Name**	***β*_**0**_(p)**	***β*_**1**_(p)**	***β*_**2**_(p)**	***β*_**3**_(p)**	**D-W**
1	Rituximab	529.49 (0.000)	19.94 (0.032)	737.89 (0.000)	8.35 (0.587)	1.95
2	Trastuzumab	838.27 (0.010)	23.36 (0.371)	760.11 (0.104)	−10.29 (0.817)	2.00
3	Bevacizumab	854.03 (0.024)	11.06 (0.715)	2,628.39 (0.000)	503.69 (0.000)	1.93
4	Sorafenib	49.81 (0.492)	5.68 (0.351)	879.97 (0.000)	12.94 (0.215)	2.10
5	Bortezomib	139.72 (0.089)	4.15 (0.538)	632.05 (0.000)	31.57 (0.009)	1.93
6	Erlotinib	548.36 (0.000)	2.05 (0.829)	1,162.27 (0.000)	−0.55 (0.973)	1.95
7	Nimotuzumab	486.04 (0.026)	−12.32 (0.466)	425.78 (0.091)	52.56 (0.094)	2.14
8	Apatinib	23.0 (0.928)	7.19 (0.728)	334.31 (0.289)	175.76 (0.000)	1.97
9	Everolimus	−4.04 (0.838)	3.23 (0.057)	33.19 (0.247)	−4.16 (0.152)	2.00
10	Recombinant human endostatin	1,718.39 (0.000)	−12.11 (0.550)	1,191.47 (0.002)	54.76 (0.121)	2.04

### Changes in Affordability of Patients

To compare the affordability of the studied anti-cancer medicines before and after medical reimbursement, we excluded chidamide, lapatinib, and abiraterone because they had zero purchase data before policy implementation. Among the remaining 12 drugs, one indication for each drug was selected to calculate the affordability. Table 6 shows the affordability during the mPFS treatment course of urban and rural patients before and after policy implementation. Before policy implementation, the affordability rates of patients were 0.64–16.02 times higher than per capita annual disposable income for urban patients and 1.50–37.76 times higher than per capita annual disposable income for rural patients. With the PAP, only fulvestrant could be affordable. After policy implementation, the affordability was obviously improved (0.06–1.90 times higher than that of per capita annual disposable income for urban patients and 0.13–4.46 times higher than that of per capita annual disposable income for rural patients). Therefore, most of the studied anti-cancer medicines could be affordable for patients.

**Table 6:** The change of patient affordability for mPFS treatment course before and after the NHIC policy.

**Name**	**Indication**	**Treatment course base on mPFS**	**Before**				**After**	
			**OOP (patient without PAP, CNY)**	**Affordability**	**OOP (patient with PAP, CNY)**	**Affordability**	**OOP (CNY)**	**Affordability**
Rituximab	Lymphoma	6.7 M	873,596	U:16.02,R:37.76	—	—	112,659	U:1.90, R:4.46
Trastuzumab	Breast cancer	1 Y	364,989	U:6.69, R:15.78	156,424	U:2.87,R:6.76	25,218	U:0.43, R:1.00
Bevacizumab	Colorectal cancer	6.1M	237,900	U:4.36, R:10.28	156,000	U:2.86,R:6.74	18,282	U:0.31, R:0.72
Sorafenib	Kidney cancer	5.7 M	285,001	U:5.23, R:12.32	150,000	U:2.75,R:6.48	27,770	U:0.47, R:1.10
Bortezomib	Mantle cell lymphoma	14.9 M	645,213	U:11.83,R:27.89	215,071	U:3.94,R:9.30	62,488	U:1.58, R:3.71
Erlotinib	Non-small cell lung cancer	7.5 M	103,500	U:1.90, R:4.47	55,200	U:1.01,R:2.39	8,775	U:0.15, R:0.35
Nimotuzumab	Nasopharynx cancer	7 M	204,204	U:3.74, R:8.83	—	—	20,420	U:0.52, R:1,21
Apatinib	Gastric adenocarcinoma	2.6 M	57,045	U:1.05, R:2.47	—	—	7,213	U:0.06, R:0.14
Fulvestrant	Breast cancer	4.6 M	34,798	U:0.64, R:1.50	16,312	U:0.30,R:0.71	2,199	U:0.06, R:0.13
Everolimus	Kidney cancer	4.9 M	72,765	U:1.33, R:3.15	—	—	8,702	U:0.22, R:0.52
Lenalidomide	Multiple myeloma	10M	631,338	U:11.58,R:27.29	—	—	49,589	U:1.25, R:2.94
Recombinant human endostatin	Non-small cell lung cancer	6 M	97,949	U:1.80, R:4.23	—	—	12,852	U:0.33, R:0.76

*Before the NHIC policy intervention, the per capita annual disposable income was CNY 54,538 in urban area and CNY 21,333 in rural area; after the NHIC policy intervention, the per capita annual disposable income was CNY 59,308 in urban area and CNY 25,263 in rural area*.

*U, Urban; R, Rural*.

*M, Months; Y, Year*.

## Discussion

### Decrease in the Price of Innovative Anti-cancer Medicines

The high cost of medical treatment has been a long-term problem in China. Expensive anti-cancer medicines prevent patients, especially low-income individuals, from seeking medical treatment. Thus, reducing the price of medicines is needed.

The NHIC policy stipulates that the governments should use their bargaining power to effectively reduce the high price of innovative anti-cancer medicines. The price of trastuzumab has decreased by 65.45% (from a daily cost of CNY 999.97 to CNY 345.45) after a series of price negotiations. The policy has significantly reduced the economic burden on patients. However, the discovery and research of innovative medicines are complex and lengthy processes. Pharmaceutical companies invest a lot of talent and financial resources in drug research and development; therefore, it is important to protect innovative profits. The centralised strategic price negotiation underpinned by evidence from Health Technology Assessment (HTA) panels were drawn from a pool of pharmacoeconomists and health insurance auditors to guarantee corporate innovation profits. Pharmaceutical companies that take part in price negotiations could then obtain benefits, such as bulk sales, after their drugs were included in the medical insurance catalogue list. The government vigorously supported the development and production of innovative medicines under the guidance of the Health China Strategy and the policy established by an innovative country. Thus, enterprises should break through technical problems and continue to promote the research and development of innovative drugs under the guidance of the spirit of innovation. The NHIC policy benefits people, and pharmaceutical companies have received widespread attention from society because of this. Entering the medical insurance catalogue is a positive incentive for pharmaceutical manufacturers to speed up research and development. The price drop of innovative anti-cancer medicines is also conducive to relieving pressure on medical insurance funds.

The government has implemented a “zero-tariff” policy to ensure accessibility and promote the price reduction of imported innovative anti-cancer medicines. However, the number of anti-cancer medicines in China is relatively lower than that in other countries, with most anti-cancer medicines in the country being generic drugs, because of patent protection and technology monopolies. Hence, the government should increase the subsidy for domestic pharmaceutical companies, cultivate pharmaceutical talents, and speed up the approval process of innovative drugs to embrace the innovative enthusiasm of enterprise in China.

### Positive Effects of the NHIC Policy on the Availability of the Studied Drugs

The availability of innovative anti-cancer medicines is an important index to evaluate whether patients could access medical treatment. This study found that patients had more chances to access the studied anti-cancer medicines in tertiary hospitals than in secondary hospitals throughout the study period. This could be due to the fact that patients in China are more inclined to seek medical treatment in tertiary hospitals when they have serious illnesses, such as cancer. However, an increase in the number of patients with cancer will greatly increase the resource demand. The mean availability of the studied anti-cancer medicines was 27.44%, implying that these drugs were hardly available in the surveyed hospitals before the NHIC policy was implemented, and this low availability may be caused by various factors. One factor is the medication expenditures related to the availability of these drugs in hospitals in LMICs (50). The 15 negotiated innovative anti-cancer medicines were all originator brands, and the prices of these drugs were higher than those of other common medicines even after the price negotiation. The high cost might be related to the low availability of these medicines in hospitals. According to the study of Zhu et al. in Jiangsu province, health service providers make higher profits from more costly pharmaceuticals because of the markup ratio prescribed by the government. The “zero-tariff” policy achieved full promotion in the public hospitals in Jiangsu province by the end of 2015. However, for hospitals, capital occupation costs, management costs, and damage costs are expensive, which are likely to reduce the revenue of hospitals without the mark-up ratio for high-value drugs. Therefore, due to financial constraints, secondary hospitals were unable to supply the innovative anti-cancer medicines (18). Furthermore, a study conducted in Pakistan showed that government hospitals experienced medicine shortage or medicine unavailability more frequently compared to private hospitals (17). In addition, some of the negotiated drugs lack real-world data, and similar alternative medicines are being sold in hospitals; thus, the purchase enthusiasm was not high.

After policy implementation, the mean availability increased to 43.33%, which means that the 15 innovative anti-cancer medicines became available in a few of the surveyed hospitals. Thus, the NHIC policy had a positive impact on the availability of medications in the hospital. However, drug availability is still low, which might be associated with the control of medical insurance cost. The control of medical insurance cost is an effective way to control the growth of medical expenses by allocating the medical insurance budget to all medical institutions and covering all medical services. Once the medical insurance costs of the hospital exceed the budget, however, the hospital needs to pay the excess cost. As the price of innovative anti-cancer medicines remained high, the hospitals were not motivated to pay the bill.

Hospitals should insist on the rational use of innovative anti-cancer medicines to avoid unnecessary medical expenses. Moreover, the government should release a supporting policy to separate the cost of innovative anti-cancer medicines and innovate the health insurance payment of negotiated drugs to facilitate the availability of innovative anti-cancer medicines in hospitals.

### Factors Associated With Changes in Utilisation

The epidemic characteristics of cancer affect the utilisation of drugs. Lung cancer and breast cancer have a high incidence in China; the baseline levels of anti-cancer treatment drugs such as trastuzumab, bevacizumab, and recombinant human endostatin were high. The monthly utilisation of 10 of the study medicines suddenly increased in September 2017. One possible explanation is that new patients and their doctors waited for the implementation of the policy to use much cheaper drugs. The utilisation of innovative anti-cancer medicines increased at different levels after the policy was implemented. The most important reason was the price reduction, which may have affected the purchasing behaviour of patients. Many factors were associated with the changes in utilisation. For example, the remarkable increase in the utilisation of bevacizumab and bortezomib may be related to the cancellation of the PAP, which possibly led patients to buy more drugs at their own expense. The utilisation of apatinib for gastric adenocarcinoma significantly also increased, mainly because gastric adenocarcinoma had a high incidence in China. Furthermore, there was no obvious upward trend in the utilisation of other innovative anti-cancer medicines, which may be associated with the limitation of indications by the insurance program. For example, only eight treatment courses of rituximab, which is used to treat relapsed or drug-resistant follicular central lymphoma and non-Hodgkin's lymphoma, are covered by medical insurance. In Nanjing, patients with cancer can purchase innovative anti-cancer medicines with a prescription from a physician at the specialty pharmacy.

This study analysed the utilisation of innovative anti-cancer medicines in hospitals but not in retail pharmacies. Therefore, this study cannot completely reflect the utilisation of innovative anti-cancer medicines in Nanjing. In addition, the off-label drug use of innovative anti-cancer medicines has been previously reported. Standardised rational medication in hospitals has improved with the development of guidelines for the clinical diagnosis and treatment of cancers. This improvement may affect the utilisation of innovative anti-cancer medicines.

Including innovative anti-cancer medicines in the medical insurance implies an increase in insurance expenditures in the future and calls for a more careful monitoring of drug use. The government should improve the dynamic adjustment mechanism of the medical insurance catalogue to guarantee the accessibility of patients to innovative drugs and reduce the burden of medical insurance funds.

### Improvement in the Affordability of Innovative Anti-cancer Medicines

Before the implementation of the NHIC policy, the OOP expenditures on the 12 innovative anti-cancer medicines for the mPFS treatment course were much higher than the per capita annual disposable income per capita. Even when the PAP relieved the economic burden of six innovative anti-cancer medicines to some degree, the high cost of innovative anti-cancer medicines may impoverish more households and lead to more premature cancer deaths (51). Therefore, the drug price reduction is closely related to winning the battle against poverty. Drug price reduction and reimbursement effectively alleviated most of the financial burden on patients. With these, patients can purchase innovative anti-cancer medicines, which can effectively improve the quality and length of life.

However, although the NHIC policy improved drug affordability, the supporting policies that help reduce the financial burden of innovative anti-cancer medicines were inadequate. For example, the PAP was cancelled after the NHIC policy was implemented. However, the affordability problem was even more serious for rural patients than for urban patients, especially low-income patients, during the study period. Hence, the government should increase tax incentives for medicines and encourage companies to implement more assistance projects to promote the PAP and improve the medical security system of China. Moreover, patients need to undergo a number of medical examinations and standard tests for cancer before being diagnosed. They also need to use adjuvant chemotherapy drugs for treatment. However, the costs of medical examinations and adjuvant chemotherapy are a tremendous financial burden.

Policies should focus on addressing the inequity in the affordability of innovative anti-cancer medicines between urban and rural areas. The government should also consider including medical examinations and adjuvant chemotherapy in the medical insurance coverage and reducing the ratio of individual payments. Setting the reimbursement ratio based on the actual conditions of patients is a better measure; the reimbursement ratio of rural patients should be higher than that of urban patients. Moreover, the government should speed up the progress of medical insurance negotiations to include more innovative anti-cancer medicines in medical insurance to reduce the financial burden of more patients with cancer. With the increase in the incidence of cancer, the government should increase investment in health. Early screening, detection, diagnosis, and treatment are of great importance to protect the health of the people.

Our study has several strengths. This study is one of the few quantitative studies that provide evidence on the accessibility of the 15 negotiated innovative anti-cancer medicines. The study findings will serve as a basis for policymakers in the government to optimise and improve supporting policies. This study selected 36 months of data before and after the policy was implemented to comprehensively evaluate the long-term effects of the NHIC policy. The ITS model was used to better reflect the impact of policy interventions and the later stages of intervention. Nevertheless, this study has some limitations. First, we underestimated the availability and utilisation of anti-cancer medicines because private hospitals and pharmacies were not included in this study. Second, this study lacked a control group to explore other factors besides policy implementation that resulted in changes in drug utilisation. Third, this study was only conducted in Nanjing, Jiangsu province. More municipalities and provinces should be included to increase the representativeness of the findings. Medication equity is one of the purposes of the NHIC policy. Changes in medication equity after policy implementation may need to be analysed in future studies.

## Conclusion

This study revealed that the NHIC policy effectively reduced the price and improved the availability and utilisation of 15 negotiated innovative anti-cancer medicines. The NHIC policy reduced the economic burden of patients with cancer. However, the availability of these innovative anti-cancer medicines remains low. The utilisation rate of innovative anti-cancer medicines is affected by many factors, which include the incidence of cancer, limitation of indications within the insurance program, and the rational use of innovative anti-cancer medicines. Improving supporting policies is necessary to promote the use of innovative anti-cancer medicines, and reducing the financial burden of patients is closely related to winning the battle against poverty. The government should introduce more policies to reduce the financial burden of patients and improve the health level of people. In addition, more innovative anti-cancer medicines should be included in the national price negotiation. The government should consider including medical examinations and adjuvant chemotherapy in the medical insurance and increase investment in health.

## Data Availability Statement

The original contributions presented in the study are included in the article/supplementary material, further inquiries can be directed to the corresponding authors.

## Author Contributions

WF, XX, and XL initiated the study concept. WF conducted data analysis and wrote the first draft of the manuscript. XX and YZ collected the drugs clinical trials data. HD led the data collection. XL and LS contributed to the data analysis and interpretation of the data and revised the draft of the manuscript. All authors read and approved the final manuscript.

## Conflict of Interest

The authors declare that the research was conducted in the absence of any commercial or financial relationships that could be construed as a potential conflict of interest.

## Publisher's Note

All claims expressed in this article are solely those of the authors and do not necessarily represent those of their affiliated organizations, or those of the publisher, the editors and the reviewers. Any product that may be evaluated in this article, or claim that may be made by its manufacturer, is not guaranteed or endorsed by the publisher.
